# Population structure analysis on 2504 individuals across 26 ancestries using bioinformatics approaches

**DOI:** 10.1186/1471-2105-16-S15-P19

**Published:** 2015-10-23

**Authors:** Jing Wang, David C Samuels, Yu Shyr, Yan Guo

**Affiliations:** 1 Center for Quantitative Sciences, Vanderbilt University School of Medicine, Nashville, TN 37232, USA; 2 Center for Human Genetics Research, Vanderbilt University, Nashville, TN 37232, USA

## Background

Characterizing genetic diversity is crucial for reconstructing human evolution and for understanding the genetic basis of complex diseases; however, human population genetics are very complicated. Previously, we proved that based on the Hardy-Weinberg equilibrium, the heterozygous vs. non-reference homozygous single nucleotide polymorphism (SNP) ratio (het/nonref-hom) is two[1]. Later, we found that this ratio is race dependent, with African being the most genetically diverse race and Asian being the most homozygous[2]. This observation prompted us to conduct further study to understand the reasoning behind this diversity.

## Materials and methods

Using the 1000 Genomes Project (1000G) released genomic data of 2504 individuals (26 races from five major-races), we first computed the (het/nonref-hom) ratio which has been applied as a quality control parameter for sequencing data[1,3].

## Results

**Figure 1:**
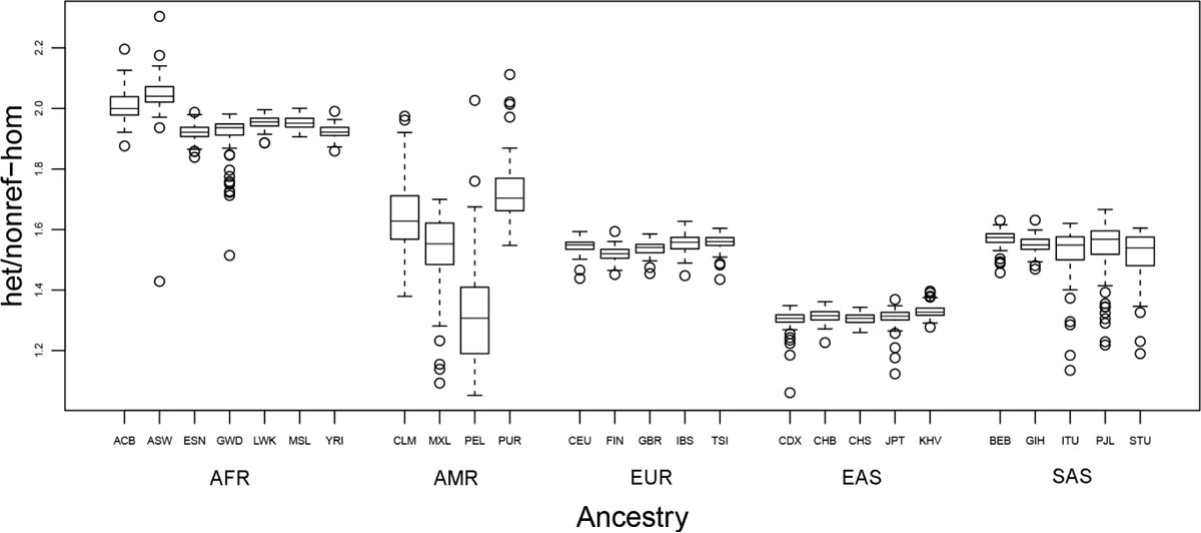
het/nonref-hom ratio across 26 ancestries.

**Figure 2:**
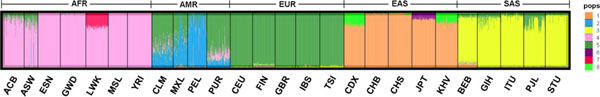
Population structure inferred from the 1000G genetic data.

As expected, we found that the het/nonref-hom ratio is strongly associated with human ancestry. Africans had the highest het/nonref-hom ratios, followed by Americans and Europeans, and East Asians had the lowest (Figure 1). More interestingly, the het/nonref-hom ratios of South Asians are much higher than those of East Asians, and Americans showed the highest range (Figure 1). Thus we further quantitatively analyzed genetic variation in human populations on the 1000G dataset of 10^11^ observed genotypes (2504 individuals at 13424776 SNPs) using Structure 2.3.4[4]. The resulting population structure is consistent with the major geographical regions. All races identified a dominate origin population, except Americans who had the most variation in the structure, represented by several populations including the dominant population of Europeans (Figure 2). Moreover, East Asians and South Asians were found to originate from different ancestries (Figure 2).

## Conclusions

Using novel bioinformatics approach, we identified new insights into the history and geography of human evolution, and are valuable for tracking human migration and adaptation to local conditions.

## References

[B1] Guo Y Ye F Sheng Q Clark T Samuels DC Three-stage quality control strategies for DNA re-sequencing data Brief Bioinform 2013 10.1093/bib/bbt069 PMC4492405 24067931

[B2] Wang J Raskin L Samuels DC Shyr Y Guo Y Genome measures used for quality control are dependent on gene function and ancestry Bioinformatics 2015 31 3 318 323 25297068 10.1093/bioinformatics/btu668 PMC4308666

[B3] Guo Y Zhao S Sheng Q Ye F Li J Lehmann B Pietenpol J Samuels DC Shyr Y Multi-perspective quality control of Illumina exome sequencing data using QC3 Genomics 2014 103 5-6 323 328 24703969 10.1016/j.ygeno.2014.03.006 PMC5755963

[B4] Hubisz MJ Falush D Stephens M Pritchard JK Inferring weak population structure with the assistance of sample group information Mol Ecol Resour 2009 9 5 1322 1332 21564903 10.1111/j.1755-0998.2009.02591.x PMC3518025

